# Comparative Genomic Analysis of Ochratoxin A Biosynthetic Cluster in Producing Fungi: New Evidence of a Cyclase Gene Involvement

**DOI:** 10.3389/fmicb.2020.581309

**Published:** 2020-12-18

**Authors:** Massimo Ferrara, Antonia Gallo, Giancarlo Perrone, Donato Magistà, Scott E. Baker

**Affiliations:** ^1^Institute of Sciences of Food Production (ISPA), National Research Council (CNR), Bari, Italy; ^2^Institute of Sciences of Food Production (ISPA), National Research Council (CNR), Lecce, Italy; ^3^Functional and Systems Biology Group, Environmental Molecular Sciences Division, Pacific Northwest National Laboratory, Richland, WA, United States; ^4^DOE Joint Bioenergy Institute, Emeryville, CA, United States

**Keywords:** ochratoxin A genes, *Aspergillus*, *Penicillium*, genomic analysis, SnoaL domain, cyclase

## Abstract

The widespread use of Next-Generation Sequencing has opened a new era in the study of biological systems by significantly increasing the catalog of fungal genomes sequences and identifying gene clusters for known secondary metabolites as well as novel cryptic ones. However, most of these clusters still need to be examined in detail to completely understand the pathway steps and the regulation of the biosynthesis of metabolites. Genome sequencing approach led to the identification of the biosynthetic genes cluster of ochratoxin A (OTA) in a number of producing fungal species. Ochratoxin A is a potent pentaketide nephrotoxin produced by *Aspergillus* and *Penicillium* species and found as widely contaminant in food, beverages and feed. The increasing availability of several new genome sequences of OTA producer species in JGI Mycocosm and/or GenBank databanks led us to analyze and update the gene cluster structure in 19 *Aspergillus* and 2 *Penicillium* OTA producing species, resulting in a well conserved organization of OTA core genes among the species. Furthermore, our comparative genome analyses evidenced the presence of an additional gene, previously undescribed, located between the polyketide and non-ribosomal synthase genes in the cluster of all the species analyzed. The presence of a SnoaL cyclase domain in the sequence of this gene supports its putative role in the polyketide cyclization reaction during the initial steps of the OTA biosynthesis pathway. The phylogenetic analysis showed a clustering of OTA SnoaL domains in accordance with the phylogeny of OTA producing species at species and section levels. The characterization of this new OTA gene, its putative role and its expression evidence in three important representative producing species, are reported here for the first time.

## Introduction

Next-generation sequencing (NGS) technologies have opened a new era in the study of biological systems by significantly increasing the catalog of fungal genomes sequences available for analysis. The Fungal Genomics Program^1^ of the United States Department of Energy (DOE) Joint Genome Institute (JGI) has partnered with the international scientific community to support genomic exploration of the fungal kingdom and to help in addressing this lack of knowledge (Grigoriev et al., 2011). The exponential growth of data resulting from several large-scale genomics initiatives, like the 1000 Fungal Genomes Project^2^ and the *Aspergillus* Whole Genus Sequencing Project (Vesth et al., 2018; Kjærbølling et al., 2020, 2018), have provided novel genomic information of a vast amount of fungal species. In parallel with increased genome sequencing throughput, transcriptomic data from fungi have become easily attainable with RNA-seq approaches enabling current gene modeling pipelines to combine genome sequences with deep transcript sequencing data. This integrated approach has led to improvements in genome annotation and *de novo* gene model generation, allowing the identification of previously “unseen” genes that are often comparatively small in size and missed by prior automated annotation pipelines (Grigoriev et al., 2014). This revolutionary approach to genome characterization has enabled a large-scale *in silico* identification of putative genes encoding novel enzymes, metabolic pathways, and bioactive compounds.

Secondary metabolites (SMs) produced by microorganisms are of major interest because of their suitability for the treatment of infectious diseases, as immunosuppressants, or as sources of new and innovative therapeutic agents; however, they are also implicated in food safety and human health as mycotoxins or bacterial toxins, or antibiotics (Brakhage and Schroeckh, 2011; Frisvad and Larsen, 2015). During the 1990s, a variety of SMs were genetically characterized and the clustered arrangement of genes involved in the biosynthesis of a single SM was studied (Sidhu, 2002). Gene cluster discovery in fungi was complex and time-consuming in the pre-genomic era, since their study involved cumbersome traditional molecular methods. Conversely, in the genomic era, the investigations of genome encoded biosynthetic potential have accelerated and the potential for exploitation of biosynthetic gene clusters gained a great attention.

Comparative analysis of predicted genes has led to the identification of many SM clusters of known metabolites, revealing the genetic basis of their biosynthesis and regulation (Kjærbølling et al., 2018). On the other hand, the same approach has led to the prediction of novel cryptic clusters for still unknown microbial metabolites, shedding light on new biosynthetic pathways never explored before (Khaldi et al., 2010; Metzker, 2010; Cacho et al., 2014; de Oliveira and Ocaña, 2015). However, most of the clusters identified by genome analysis require more investigation before we completely understand the pathway steps and the regulatory network behind the metabolite biosynthesis (Brakhage, 2013).

An appropriate example for how the genomic approach has led to the identification and characterization of biosynthetic gene clusters (BGC), is ochratoxin A (OTA), primarily identified in *Aspergillus niger*, and subsequently in *A. carbonarius* (Pel et al., 2007; Baker et al., 2009). Ochratoxin A is a well-known mycotoxin with wide distribution on food and feed, including cereal products, grapes and by-products, coffee, beverages, cocoa, nuts, dried fruits, and cured meat (Jørgensen, 2005; el Khoury and Atoui, 2010). Ochratoxin A is nephrotoxic, hepatotoxic, embryotoxic, teratogenic, neurotoxic, immunotoxic, genotoxic, and carcinogenic with species and sex-related differences (IARC, 1993; Castegnaro et al., 2006; Malir et al., 2016).

The analysis of *A. carbonarius* genomic data revealed the key role of three genes (*AcOTApks, AcOTAnrps*, and *AcOTAhal*) in the OTA biosynthesis (Gallo et al., 2012, 2017; Ferrara et al., 2016), and the involvement of two genes encoding for a cytochrome P450 oxidase and a bZIP transcription factor has been demonstrated recently in six different OTA producing species (Wang et al., 2018). This evidence, supported by molecular genetic approaches, has defined the role of these five core genes as essential for OTA biosynthesis. Moreover, a comparative analysis of OTA-biosynthetic clusters in *A. steynii, A. westerdijkiae, A. niger, A. carbonarius*, and *P. nordicum* has revealed substantial synteny in cluster organization (Gil-Serna et al., 2018).

To date, OTA is known to be produced by several species of genus *Aspergillus* – more than twenty mainly within Sect. *Nigri* and *Circumdati*, and at least three *Penicillium* species (*P. nordicum, P. thymicola, P. verrucosum*). The recent availability in JGI Mycocosm and/or GenBank of several new genome sequences within *Aspergillus* and *Penicillium* OTA producer species has encouraged us to extend the analysis of cluster organization to 21 OTA producing species, 19 *Aspergillus* and 2 *Penicillium*. The extensive analysis of the genomic region related to OTA cluster genes, indicated presence of an additional previously undescribed gene, with a “SnoaL-like cyclase” domain located between the polyketide synthase (PKS) and non-ribosomal peptide synthase (NRPS)genes.

The role of cyclization in polyketide biosynthesis is complex and important. There is limited data regarding the role of specific cyclase genes in secondary metabolite biosynthesis. More often, cyclization is thought to happen either spontaneously via intramolecular interactions within the growing polyketide or via cyclization domains that are part of the PKS; alternative hypothesis could be the involvement of an external cyclase gene respect to the PKS domain structure.

In this respect, the characterization of this SnoaL-like cyclase gene, its phylogenetic relationships, putative role and expression evidence in three important representative species under producing conditions are reported for the first time in this manuscript.

## Materials and Methods

### Ochratoxin Cluster Analysis in Ochratoxigenic Fungi

The analysis of the complete OTA cluster of producing species was carried out by retrieving sequence information and gene annotation from JGI Genome Portal^3^ for *A. sclerotioniger* CBS115572 v1.0, *A. carbonarius* ITEM 5010 v3.0, *A. niger* ATCC 13496 v1.0, *A. steynii* IBT 23096 v1.0, *A. muricatus* CBS 112808 v1.0, *A. roseoglobulosus* CBS 112800 v1.0, *A. flocculosus* CBS 112785 v1.0, *A. pulvericola* CBS 137327 v1.0, *A. elegans* CBS 116.39 v1.0, *A. welwitschiae* CBS139.54b v1.0, *A. affinis* CBS 129190 v1.0, *A. cretensis* CBS 112802 v1.0, *A. subramanianii* CBS 138230 v1.0, *A. albertensis* IBT 14317 v1.0, *A. alliaceus* CBS 536.65 v1.0, *A. sulphureus* (*fresenii*) CBS 550.65 v1.0, *A. sclerotiicarbonarius* CBS 121057. For *A. westerdijkiae*, and *P. nordicum*, we considered the gene annotation reported by other authors (Gil-Serna et al., 2018; Wang et al., 2018) and compared it with the data available at JGI for *A. westerdijkiae* CBS 112803 v1.0 and *P. nordicum* DAOMC 185683 v1.0. Sequence information for *A. ochraceus* FC-1 (GCA_004849945.1) and *P. verrucosum* BFE808 (GCA_000970515.2) were retrieved from NCBI database. For each species the main characteristics of the biosynthetic genes were described including the information related to DNA, mRNA and proteins. Gene features and annotations were retrieved by BlastX and manually cured, when not available.

### Fungal Strains and Growth Conditions

Fungal strains used were *A. carbonarius* ITEM 5010, *A. westerdijkiae* ITEM 9607 and *P. nordicum* ITEM 9634 from the Agro-Food Microbial Culture Collection of the Institute of Sciences of Food Production, CNR, Bari, Italy^4^. Fungal strains were routinely grown on PDA (Oxoid, United Kingdom).

For confirmation of PKS cyclase gene (*otaY*) expression under OTA permissive condition, 100 μl of a conidial suspension (10^6^ conidia/ml) of each strain were inoculated on yeast extract sucrose (YES) agar plates (yeast extract 20 g/l, sucrose 150 g/l, agar 20 g/l) overlaid with sterile cellophane membranes. Incubation was carried out in the dark at 25°C. Fungal mycelium was harvested after 5 days post inoculation (dpi), then frozen in liquid nitrogen and stored at −80°C for RNA extraction. Triplicate cultures were prepared and analyzed for each strain.

### Nucleic Acid Extraction, cDNA Synthesis, RT-PCR and Sequencing

Genomic DNA (gDNA) was extracted using the Purelink Genomic Plant DNA kit (Invitrogen, San Diego, CA). Total RNA was extracted from frozen mycelium ground in liquid nitrogen using the RNeasy kit (Qiagen, Hilden, DE) according to the manufacturer’s protocol. Any possible DNA contamination was removed by RNase-free DNase I (Qiagen) digestion. RNA aliquots were stored at −80°C. First-strand cDNA was synthesized using 1 μg of total RNA with SuperScript IV First-Strand Synthesis System (Invitrogen, San Diego, CA) according to the manufacturer’s protocol. Reverse transcription-PCR (RT-PCR) was used to analyze the expression of *otaY* transcripts in *A. carbonarius*, *A. westerdijkiae* and *P. nordicum* strains. Amplifications were carried out on gDNA and cDNA using the Platinum SuperFi II PCR Master Mix (Invitrogen, San Diego, CA). The primer pairs listed in Table 1 were used under the following conditions: 98°C for 30 s, followed by 35 cycles of 98°C for 10 s, 60°C for 10 s, and 72°C for 30 s, followed by a final extension step at 72°C for 5 min. Amplicons were checked by 1.5% (w/v) agarose gel electrophoresis and sequenced using the BigDye Terminator v3.1 Cycle Sequencing Kit (Applied Biosystem, United States).

**TABLE 1:** Primer sequences for RT-PCR.

Primer name	Nucleotide sequence
Aspca_cycl_F	5′-ACCATCCTCACCACCCTTGT-3′
Aspca_cycl_R	5′-CCGTACTCCTCACCACCAA-3′
Aspwst_cycl_F	5′- TGGATCTCAAATCTCGCGCT-3′
Aspwst_cycl_R	5′- CCTCCCTTCTCTCTCCACCT-3′
Pnord_cycl_F	5′- TCCCGTGCTCAACTGTTTCT-3′
Pnord_cycl_R	5′- TCTTCTCGTCCTCGGGATGT-3′

The deduced amino acid sequences were determined using the EMBOSS Sixpack tool^5^. Protein sequences were aligned by using muscle algorithm and the functional domain was detected by SMART^6^ analysis.

### Comparative Genome Analysis for Cyclases in Fungi

During the analysis of genomes sequenced as part of the Whole Aspergillus Genus Sequencing Project, gene clusters predicted to encode the enzymes involved in OTA biosynthesis were identified in several of the genomes. Within those clusters, we noted the conserved presence of a putative SnoaL domain containing cyclase. In some of the previously published *Aspergillus* genomes, the cyclase gene was present, but unidentified by prior versions of the gene modeling software. In these cases, the cyclase gene was manually curated. Following the discovery of the SnoaL domain containing cyclase we searched all available *Aspergillus* genomes for SnoaL cyclases and downloaded all those in the range of 160–230 amino acids to perform phylogenetic analysis.

### Multiple Sequence Alignment and Phylogenetic Analysis of Cyclase Domain

A total of 863 amino acidic sequences of SnoaL domains were retrieved from *Aspergillus* fungal genomes portal (MycoCosm, DOE-JGI) and subsequently aligned using the MAFFT software Version 7 (Katoh and Standley, 2013). The phylogenetic analysis of all the SnoaL domains was conducted by the Maximum Likelihood (ML) using RaxML hybrid code (Stamatakis, 2014) at the CIPRES Science Gateway (Miller et al., 2010). Bootstrapping was performed to assess the robustness of the phylogeny. The most robust approach to infer phylogenetic relationship was ML analysis by using the RAxML algorithm, setting the bootstrap analysis to 1000 runs, the GAMMA Model parameters were used up to an accuracy of 0.1000000000 Log Likelihood units.

For our OTA-specific phylogenetic study, we selected the 23 OTA clustered SnoaL cyclases out of a total of 863 from our preliminary analysis (Supplementary Figure 1). We also included two SnoaL cyclases from non-OTA strains of *A. parvulus*, *A. cervinus*. Finally, we included a SnoaL cyclase from *Metarhizium robertsii* found by genomic analysis to belong to a putative-partial OTA-like cluster. For this analysis the aa sequences domains were aligned both with Clustal and MUSCLE alignment in MegaX software (Kumar et al., 2018) by trimming manually the external not well aligned part of the obtained alignment; the validity and the robustness of the alignment was checked on T-Coffe server^7^. The best substitution model was calculated in Mega X. The evolutionary history was inferred by using the Maximum Likelihood method and JTT matrix-based model (Jones et al., 1992) by the use of Gamma distribution and Invariant sites. There were a total of 115 positions in the final dataset. Evolutionary analyses were conducted in MEGA X. Initial tree(s) for the heuristic search were obtained automatically by applying Neighbor-Join and BioNJ algorithms to a matrix of pairwise distances estimated using a JTT model, and then selecting the topology with superior log likelihood value. A discrete Gamma distribution was used to model evolutionary rate differences among sites [5 categories (+G, parameter = 1.3800)]. The rate variation model allowed for some sites to be evolutionarily invariable ([+I], 12.17% sites). The same set of alignments was converted in nexus format and run on MrBayes software (Ronquist et al., 2012) to conduct the Bayesian Posterior Probability analysis for the evolutionary tree. The Dayhoff substitution rate was set with 4 categories of Gamma distribution with invariable sites uniform distributed, the number of sequences were 26 and 126 characters with 113 unique site patterns. This analysis involved 26 amino acid sequences. All positions containing gaps and missing data were eliminated (complete deletion option).

## Results

### Annotation of OTA Cluster Structure in Ochratoxigenic Species

**FIGURE 1:**
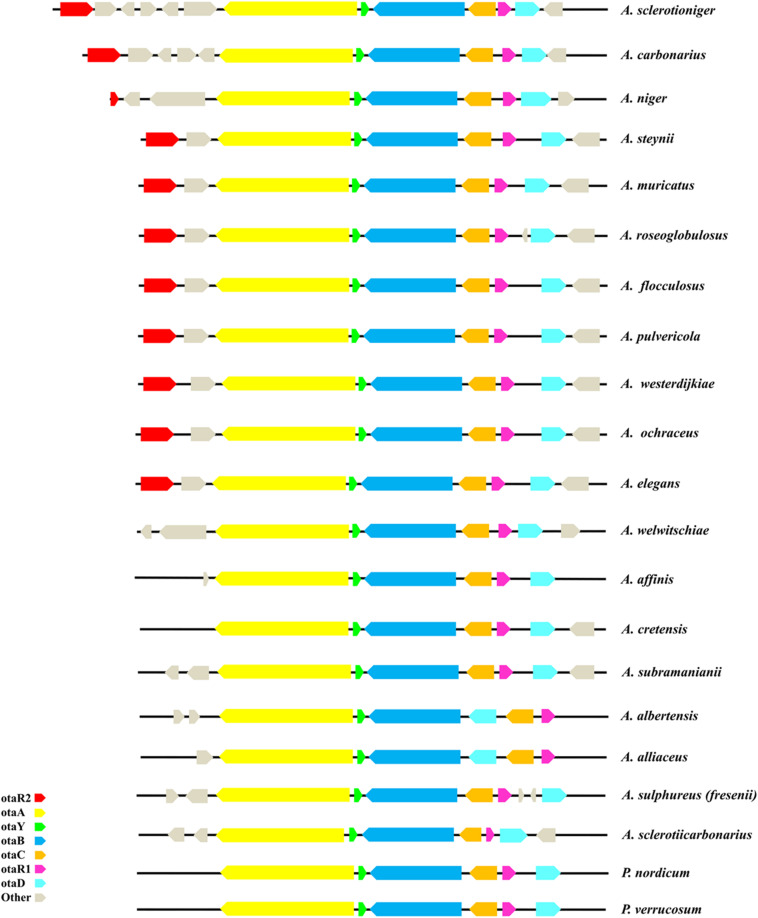
Scheme of OTA cluster in the 21 ochratoxigenic species.

Analysis of the OTA cluster was performed on 21 ochratoxigenic species, most of them belonging to the *Aspergillus* genus, with only two species belonging to *Penicillium* genus (Table 2). The length of each OTA core cluster in the species analyzed varies from 19 to 24 kbp, with differences due to variance in the intergenic space and gene model sequences. The core OTA biosynthetic cluster is composed of five genes, highly conserved in all the ochratoxigenic species. The genes encode a PKS, a NRPS, a cytochrome P450 monooxygenase, a basic leucine zipper (bZIP) transcription factor and a halogenase. All of these genes and their associated proteins have been demonstrated to be involved in the biosynthesis of OTA across a range of ochratoxigenic species (Gallo et al., 2012, 2017; Ferrara et al., 2016; Gil-Serna et al., 2018; Wang et al., 2018). Identified and characterized in this study, our genome analyses confirmed the presence of a putative OTA PKS cyclase gene in all currently sequenced OTA producing fungi. The location of the SnoaL cyclase between PKS and NRPS encoding genes as well as its transcriptional direction relative to those genes, was also assessed. Generally, synteny and reciprocal transcription direction of the core cluster genes is conserved, as well as the number of exons for coding sequences of each gene (Figure 1).

**TABLE 2:** List of main features of the genes in OTA cluster of the 21 species analyzed.

Species (core cluster length)	Gene	DNA length(bp)	Number of exons	cDNA length (bp)	aa
*Aspergillus sclerotioniger* CBS115572	otaA	8012	9	7614	2537
	otaY	420	1	420	139
(21156 bp)	otaB	5638	2	5586	1861
	otaC	1763	6	1542	513
	otaR1	973	2	921	306
	otaD	1784	6	1485	494
*Aspergillus carbonarius* ITEM 5010	otaA	8063	9	7626	2541
	otaY	372	1	372	123
(21508 bp)	otaB	5691	2	5628	1875
	otaC	1800	6	1551	516
	otaR1	800	2	744	247
	otaD	1843	6	1497	498
*Aspergillus niger* ATCC 13496	otaA	8173	9	7665	2554
	otaY	360	1	360	119
(21670 bp)	otaB	5692	2	5643	1880
	otaC	1809	6	1530	509
	otaR1	785	2	732	243
	otaD	1762	6	1494	497
*Aspergillus steynii* IBT 23096	otaA	8048	9	7611	2536
	otaY	387	1	387	128
(23671 bp)	otaB	5689	2	5640	1879
	otaC	1822	6	1542	513
	otaR1	767	2	717	238
	otaD	1774	6	1479	492
*Aspergillus muricatus* CBS 112808	otaA	8083	9	7647	2548
	otaY	507	1	507	168
(23696 bp)	otaB	5694	2	5646	1881
	otaC	1822	6	1542	513
	otaR1	746	2	696	231
	otaD	1745	6	1479	492
*Aspergillus roseoglobulosus* CBS 112800	otaA	8058	9	7653	2550
	otaY	387	1	387	128
	otaB	5699	2	5652	1883
(23599 bp)	otaC	1839	6	1542	513
	otaR1	766	2	720	239
	otaD	1755	6	1479	492
*Aspergillus flocculosus* CBS 112785	otaA	8032	9	7602	2533
	otaY	387	1	387	128
(23434 bp)	otaB	5689	2	5640	1879
	otaC	1825	6	1545	514
	otaR1	771	2	717	238
	otaD	1774	6	1479	492
*Aspergillus pulvericola* CBS 137327	otaA	8080	9	7650	2549
	otaY	387	1	387	128
(23410 bp)	otaB	5689	2	5640	1879
	otaC	1824	6	1545	514
	otaR1	766	2	717	238
	otaD	1774	6	1479	492
*Aspergillus westerdijkiae* CECT 2948	otaA	8095	9	7608	2535
	otaY	387	1	387	128
(24204 bp)	otaB	5701	2	5652	1883
	otaC	1821	6	1545	514
	otaR1	767	2	717	238
	otaD	1776	6	1479	492
*Aspergillus ochraceus* FC-1	otaA	8143	9	7656	2551
	otaY	387	1	387	128
(24204 bp)	otaB	5701	2	5652	1883
	otaC	1827	6	1545	514
	otaR1	767	2	717	238
	otaD	1776	6	1479	492
*Aspergillus elegans* CBS 116.39 v1.0	otaA	8048	9	7611	2536
	otaY	387	1	387	128
(23679 bp)	otaB	5689	2	5640	1879
	otaC	1822	6	1542	513
	otaR1	767	2	717	238
	otaD	1776	6	1479	492
*Aspergillus welwitschiae* CBS139.54b	otaA	8161	9	7653	2550
	otaY	360	1	360	119
(21674 bp)	otaB	5692	2	5643	1880
	otaC	1811	6	1530	509
	otaR1	806	2	753	250
	otaD	1762	6	1494	497
*Aspergillus affinis* CBS 129190	otaA	8096	9	7632	2543
	otaY	390	1	390	129
(23387 bp)	otaB	5692	2	5643	1880
	otaC	1833	6	1545	514
	otaR1	767	2	717	238
	otaD	1759	6	1479	492
*Aspergillus cretensis* CBS 112802	otaA	8097	9	7656	2551
	otaY	390	1	390	129
(23586 bp)	otaB	5692	2	5643	1880
	otaC	1833	6	1545	514
	otaR1	767	2	717	238
	otaD	1759	6	1479	492
*Aspergillus subramanianii* CBS 138230	otaA	8094	9	7659	2552
	otaY	381	1	381	126
23730 bp	otaB	5672	2	5646	1881
	otaC	1851	6	1542	513
	otaR1	764	2	717	238
	otaD	1771	6	1479	492
*Aspergillus albertensis* IBT 14317	otaA	8093	9	7623	2540
	otaY	378	1	378	125
(22139 bp)	otaB	5682	2	5634	1877
	otaD	1746	6	1494	497
	otaC	1810	6	1539	512
	otaR1	745	2	696	231
*Aspergillus alliaceus* CBS 536.65	otaA	8093	9	7623	2540
	otaY	378	1	378	125
(22138 bp)	otaB	5682	2	5634	1877
	otaD	1746	6	1494	497
	otaC	1809	6	1539	512
	otaR1	745	2	696	231
*Aspergillus sulphureus* (*fresenii*) CBS 550.65	otaA	8081	9	7659	2552
	otaY	381	1	381	126
(23736 bp)	otaB	5694	2	5646	1881
	otaC	1847	6	1542	513
	otaR1	749	2	702	233
	otaD	1764	6	1479	492
*Aspergillus sclerotiicarbonarius* CBS 121057	otaA	7920	11	7359	2452
	otaY	348	1	348	115
	otaB	5607	3	5493	1830
(19040 bp)	otaC	827	6	561	186
	otaR1	814	2	765	254
	otaD	1744	7	1437	478
*Penicillium nordicum* DAOMC 18563	otaA	8062	9	7617	2538
	otaY	387	1	387	128
(22769 bp)	otaB	5692	2	5643	1880
	otaC	1827	6	1542	514
	otaR1	767	2	717	238
	otaD	1772	6	1479	492
*Penicillium verrucosum* BFE808	otaA	8113/8065	9	7662/7614	2553/2537
(22931 bp)	otaY	387	1	387	128
	otaB	5689	2	5640	1879
	otaC	1831	6	1545	514
	otaR1	767	2	717	238
	otaD	1772	6	1479	492

Here, we apply the conventional naming system for the genes of OTA cluster that have already been used by Wang et al. (2018). Specifically, it is based on a three-letter code “ota” followed by a capital letter representing each individual gene, that resembles the naming systems usually used for biosynthesis genes of most fungal mycotoxins and secondary metabolites. In the core cluster, the genes encoding for the key proteins PKS and NRPS are named *otaA* (pks) and *otaB* (nrps), the genes encoding for the P450 oxidoreductase and the halogenase protein are named *otaC* (p450) and *otaD* (hal), and the gene identified in this work and encoding the putative OTA PKS cyclase is named *otaY* (cyc). For *A. westerdijkiae*, a coding sequence for a predicted PKS cyclase of 128 aa was present between *otaA* and *otaB* genes in the draft annotation of strain CBS112803 at JGI, we therefore included its annotation in the data related to the strain CECT2948 (Gil-Serna et al., 2018). Moreover, the study of OTA cluster in *A. ochraceus* FC1 (Wang et al., 2018) led to the identification of a putative coding sequence for a predicted PKS cyclase of 128 aa, located between *otaA* and *otaB* genes as in the other ochratoxigenic species.

Species of *Penicillium* are also known to produce OTA. In *P. nordicum* strain DAOM 18563, we identified a gene sequence encoding a putative PKS cyclase located between *otaA* and *otaB*. The predicted OTA core cluster gene sequences of *P. verrucosum* strain BFE808 were identified based on homology analysis with a PKS cyclase gene identified between *otaA* and *otaB* as well.

Two other genes encoding a flavin adenine dinucleotide (FAD)-dependent oxidoreductase (*otaE*) and a zinc finger DNA binding protein (*otaR2*) involved in the regulation of some core genes were identified adjacent to the core cluster in *A. ochraceus* and in proximity to the *otaA* gene (Wang et al., 2018). We have found that the genome sequences of a several species encode *otaE* and *otaR2* in the same position. There are exceptions, however: for example, the genomes of *A. sclerotioniger, A. niger* and *A. carbonarius*, have the *otaR2* gene located far from the core cluster. Very few species exhibit only the *otaE* gene in the conserved position close to *otaA.* At the opposite end of the core cluster, several species have genes encoding a peptidase or hydroxylase. The characteristics of all characterized OTA clusters are summarized in Figure 1.

The predicted amino acid sequences of PKSs encoded by *otaA* genes display the characteristic domains of a highly reducing (HR)-PKS (KS, AT, DH, CMet, ER, KR, and ACP) involved in the first step of formation of the pentaketide backbone of the OTA molecule starting from acetate and malonate. Some divergent data has emerged about the length of *otaA* and other OTA genes in *A. niger* strain ATCC13496 used in this study, as compared with data reported by other authors for *A. niger* strain CBS513.88 (Pel et al., 2007; Gil-Serna et al., 2018), the first *A. niger* strain sequenced. Most of these differences are likely due to the variety of automated annotation pipelines used, in particular related to the prediction analysis of exon/intron junctions. Analysis of the strain ATCC13496 in this study led to the identification of a putative PKS cyclase in the *A. niger* species.

In *P. verrucosum* BFE808, two possible start codons were detected for the *otaA* coding sequence, to initiate translation of predicted proteins of 2553 and 2537 aa, respectively. Similarly, two possible start codons were found in the *otaA* open reading frame of *P. nordicum* also. In the case of *A. sclerotiicarbonarius* the analysis of the *otaA* gene revealed the presence of 11 exons instead of 9, as observed in the other species.

The analysis of *otaB*, *otaC*, *otaR1*, and *otaD* genes revealed that they are well conserved in terms of gene length and coding sequence structure in the analyzed species, with the exception of *A. sclerotiicarbonarius* wherein both *otaB* and *otaD* genes exhibited one additional exon compared with other species.

Finally, an unexpected and interesting result came from the analysis of OTA core cluster organization in *A. albertensis* IBT14317 and *A. alliaceus* CBS 536.65. In these two species, the *otaD* gene was found between *otaB* and *otaR1* rather than at the end of the core cluster. It should be noted that while there are slight differences in genome size and gene model content (Kjærbølling et al., 2020) in the revision of Section *Flavi* (Frisvad et al., 2019) *A. albertensis* was tentatively synonymized to *A. alliaceus*.

### Transcription Evidence of the Cyclase Gene

**FIGURE 2:**
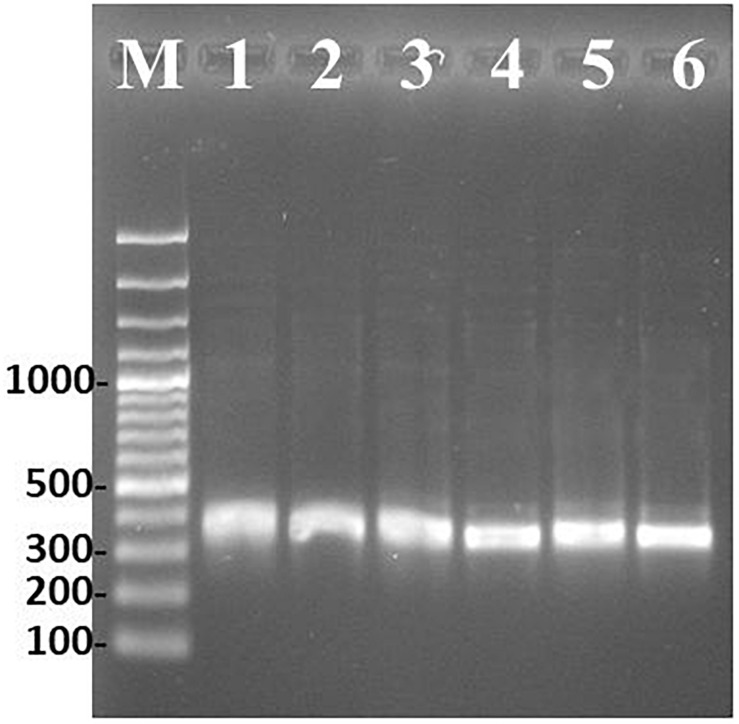
PCR amplification of cyclase gene under permissive OTA condition. Marker 100 bp (M), *A. carbonarius* ITEM 5010 gDNA (1) and cDNA (2), *P. nordicum* ITEM 9634 gDNA (3) and cDNA (4), *A. westerdijkiae* ITEM 9607 gDNA (5) and cDNA (6).

Putative OTA PKS cyclase gene transcripts were successfully amplified from gDNA and RNA extracted from *A. carbonarius* ITEM 5010, *A. westerdijkiae* ITEM 9607 and *P. nordicum* ITEM 9634, grown under OTA permissive conditions (Figure 2). Nucleotide sequences of transcripts were verified by Sanger sequencing and deposited at NCBI with accession numbers MT706047 (*A. carbonarius*), MT706048 (*A. westerdijkiae*) and MT706049 (*P. nordicum*).

**FIGURE 3:**
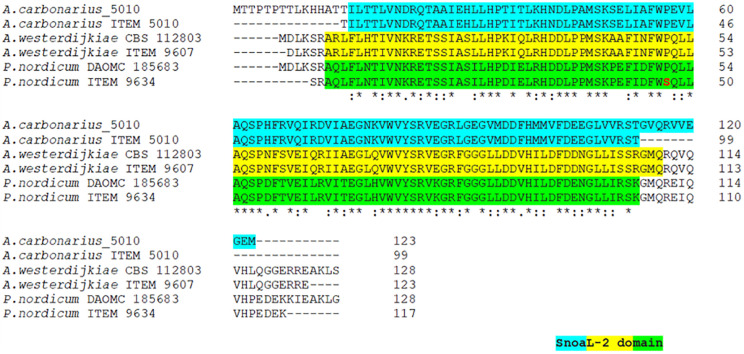
Multi-alignment of deduced cyclase amino acid sequences of *A. carbonarius* ITEM 5010, *A. westerdijkiae* ITEM 9607 and *P. nordicum* ITEM 9634 with the respective reference sequence of type strain. Amino acid substitution is reported in red. The predicted SnoaL-2 domain is highlighted in different colors for each species.

The analysis of alignment to already available reference sequences, revealed a 100% identity to the type strain sequences for *A. carbonarius* and *A. westerdijkiae*. The cyclase gene of *P. nordicum* displayed a nucleotide transition from C to T at position 131, determining an amino acid substitution from P to S. However, this substitution did not alter the SnoaL-2 functional domain prediction (Figure 3).

### Phylogeny of SnoaL Domain Genes

**FIGURE 4:**
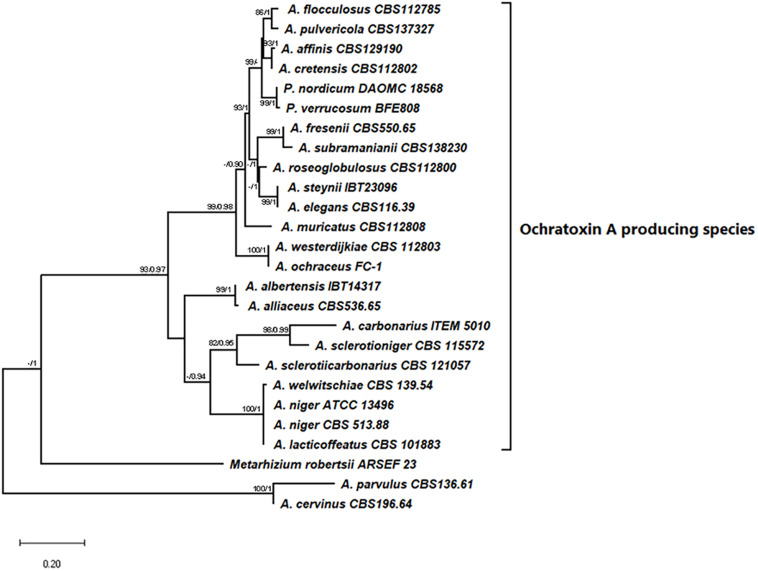
The MaxLikelihood tree of SnoaL domain with the highest log likelihood (−1837.50) is shown. Bootstrap value (>80%) and PP (>0.90) are showed next to the branch as support.

The initial phylogenetic analysis of 863 sequences of SnoaL domains from *Aspergillus* genomes, gave an alignment of 1019 distinct patterns, and the proportion of gaps and the completely undetermined characters was 84.72%. The RaxML analysis returned the tree with the highest log likelihood (−118408.77) shown in Supplementary File with bootstrap value (Supplementary Figure 1). From the analysis, all the 23 SnoaL domains identified in the OTA biosynthetic clusters, grouped together with a good support (97%) including a sister cluster of two no producing OTA species *A. cervinus* and *A. parvulus* (Supplementary Figure 1). None of the other SnoaL domains analyzed resulted related to SnoaL domains of the OTA clusters, while they formed numerous clades, sometimes well supported. On the basis of this first general phylogenetic results evidencing the robustness of OTA cyclases SnoaL domain clustering, we focused our phylogenetic analysis on the 23 cyclases from OTA biosynthetic clusters; a cyclase of *Metarhizium robertsii* found between a PKS and NRPS genes in a partial putative OTA cluster, and the cyclases of *A. parvulus* and *A. cervinus*, closely related to the group of the OTA cyclases. The phylogenetic tree resulting from the Maximum likelihood analysis with the highest log likelihood (−1837.50) is shown in Figure 4. The percentage of trees in which the associated taxa clustered together is shown next to the branches. The tree is drawn to scale, with branch lengths measured in the number of substitutions per site. On the same tree the Posterior probability of the Bayesian analysis were also reported on the branch, the Bayesian analysis give the best likelihood tree for run 1 = −1923.89 and for run 2 = −1913.05. The topology of the trees obtained by the two separate analyses is consistent with the Bayesian analysis giving higher support to the OTA cyclase cluster (Figure 4). The result of the phylogenetic analysis clearly confirmed the clustering of the OTA biosynthetic cyclases in a well-supported branch closely related to the *M. robertsii* cyclase and the two cyclase from *A. cervinus* and *A. parvulus*.

## Discussion

Next-generation sequencing technologies continue to drive a new era in mycology by greatly increasing the catalog of fungal genomes sequences available for analysis. The alacrity with which fungal genomes are being sequenced produces a vast and still-growing amount of information, which must be efficiently integrated and analyzed in order to understand enzymatic and biochemical diversity of these organisms. Despite the development of efficient bioinformatics approaches, maintaining consistency and accuracy in genome curation and annotation remains a daunting task. With each additional OTA producing fungal genome sequence produced, we perform the comparative genomic analysis of OTA biosynthetic cluster. Continuous revision and improvement of the genome annotation, and of comparative analysis of predicted genes allows for refinement and discovery that enhance our knowledge of OTA production and biochemistry. Indeed, our analyses improved the annotation of the OTA cluster in 21 producing species and led to the identification of a previously uncharacterized gene in the cluster. Whole genome analyses have led to the identification of many SM clusters of known metabolites (Kjærbølling et al., 2018), and also to the prediction of novel cryptic clusters for still unknown microbial metabolites (de Oliveira and Ocaña, 2015). But, there are still knowledge gaps associated with the OTA biosynthetic cluster that must be elucidated and new genome data and comparative analysis are critical for accurate annotation and improved biochemical understanding. In this regard, due to its widespread distribution in food, feed and beverages, OTA has been extensively studied in the areas of toxicological, contamination and exposure risks as well as the biochemistry and ecological properties of its producers. Some key enzymatic reactions of OTA biosynthesis have been defined, although the biosynthesis pathway and its regulation have not been completely elucidated. OTA is a hybrid molecule composed of a polyketide dihydroisocoumarin moiety linked via amide bond to the amino acid phenylalanine. A PKS enzyme is involved in the first step of formation of the isocoumarin group, starting from acetate and malonate, to originate the characteristic pentaketide skeleton of ochratoxin β (7-carboxy-methyl-mellein), and subsequently a NRPS catalyzes the condensation of the dihydroisocoumarin ring to phenylalanine to form OTB (ochratoxin B). Finally a halogenase enzyme is necessary for the chlorination step that transforms OTB in OTA (Gallo et al., 2012, 2014; Ferrara et al., 2016).

The overall organization of OTA core genes is well conserved among the 21 species included in this study and is in agreement with the evolutionary lineage of ochratoxigenic species. Based on the currently available genome information, our analyses of the core genes of OTA cluster revealed some differences in gene attributes probably due to different bioinformatics pipelines used for genome model prediction. Despite of these discrepancies reported by different authors, the involvement of most of these genes in the biosynthetic pathway of OTA is well established (Gallo et al., 2012, 2014; Ferrara et al., 2016; Wang et al., 2018).

We found that *A. steynii, A. muricatus, A. roseoglobulosus, A. flocculosus, A. pulvericola, A. westerdijkiae, A. ochraceus*, and *A. elegans* shared the same cluster organization with only minor differences observed in intergenic distance between *otaR1* and *otaD* genes. This group of eight species shared also the same *otaR2* gene position, located upstream of the *otaA* gene adjacent to a FAD-oxidoreductase encoding gene named *otaE* by Wang et al. (2018). In *A. sclerotioniger*, *A. carbonarius*, and *A. niger* the *otaR2* gene is also annotated upstream of the *otaA* gene, but with more than one predicted gene model located between them. The length of the coding sequence of *otaR2* gene is conserved in nearly all the above mentioned species with the exception of *A. niger* whose coding sequence is considerably shorter than in the others. In the other OTA producer species, the *otaR2* gene is not annotated but we believe that this gene has been missed in most of the species due to incomplete annotation of the genomic region adjacent to the core cluster genes. Moreover, upstream of the *otaD* gene, one to two additional genes are annotated in *A. roseoglobulosus* and *A. sulphureus*, respectively. Also, in these cases we cannot exclude that their presence needs to be confirmed by further investigations. The analysis of the OTA cluster region of *A. albertensis* and *A. alliaceus* reveals a genomic rearrangement wherein a portion of the cluster includes the genes *otaC*, *otaD*, and *otaR1*. The shift of *otaD* gene near to *otaB* gene represents a novelty never observed and described in OTA producing strains. The potential reason for the difference in gene location could be related to several events such as the presence of mobile elements in the proximity of *otaD* gene. Understanding the differences in OTA gene cluster organization merit further investigation.

Based on the genome annotation of the currently sequenced OTA producer species, we noticed that 17 out of 21 OTA producers presented a putative protein coding for a cyclase (*otaY*) between the *otaA* and *otaB* genes. This gene was not identified in the gene model catalogs of *A. welwitschiae*, *A. elegans*, *P. nordicum*, and *P. verrucosum*. However, we were able to manually identify and annotate the *otaY* sequences for inclusion in our phylogenetic analyses. Our phylogenetic analysis supports the hypothesis that the *otaY* gene is well conserved among the OTA producing species. Interestingly, the preliminary phylogenetic analysis on 863 different SnoaL domains, retrieved from all 225 *Aspergillus* genomes available in Mycocosm, indicates the clear grouping of the putative OTA cyclase genes within the same phylogenetic group, with a consistent bootstrap. The phylogenic tree showed that together with the cyclase sequences of the OTA producing strains, a sister clade closely related and well supported, comprises two species not known to produce OTA, namely *A. parvulus* and *A. cervinus*. Comparative genomic analysis of these two strains indicated that the OTA cluster was not present, although we noted that the *A. cervinus* cyclase was neighbored by uncharacterized NRPS and PKS genes. None of the other analyzed SnoaL domains appear to be related to the cyclases putatively involved in OTA biosynthesis, confirming that these latter have likely evolved together following the speciation within *Aspergillus* genus. The non-OTA associated SnoaL domain containing cyclases formed numerous clades. Thus, a phylogenetic analysis was performed on the restricted group of 23 OTA SnoaL domains (comprising 22 OTA producing species and *A. sclerotiicarbonarius* not producing OTA but displaying the complete biosynthetic cluster), a cyclase domain of *M. robertsii* in a partial OTA cluster, and the two cyclases domains of *A. parvulus* and *A. cervinus* retrieved from the previous analysis. The clustering of 23 OTA SnoaL domains was in accordance with the phylogeny at species and section level for the *Aspergilli* (Figure 4), with species of section *Nigri*, section *Flavi* and *Circumdati* were well supported. Interestingly, the cyclases of *P. verrucosum* and *P. nordicum* clustered together (bootstrap 99/1); but they belong to the well supported clade associated with *Aspergillus* Sect *Circumdati* (Figure 4). These findings support the hypothesis of a possible horizontal gene transfer between *Aspergillus* and *Penicillium* genera, particularly since it is well known that they come from a common ancestor and belong to the same family of *Aspergillaceae* (Samson et al., 2014; Houbraken et al., 2020). Moreover, previous data showed high similarity of OTA PKS between Section *Circumdati* species and *P. nordicum* (Culebras et al., 2009; Gallo et al., 2014).

In our study, we observed the occurrence of the transcript of the putative cyclase of OTA cluster in three important producing species, confirming that it was expressed during OTA biosynthesis. The characterization of nucleotide and amino acid sequences attested the presence of a domain showing high homology to SnoaLs. SnoaLs proteins have been characterized as belonging to a family of small polyketide cyclases, consisting of approximately 140 amino acids, which catalyze ring closure steps in the biosynthesis of polyketide antibiotics produced in *Streptomyces* (Sultana et al., 2004). It was suggested that the reaction catalyzed by these enzymes is an intramolecular aldol condensation, by using acid–base chemistry rather than covalent or metal ion catalysis. Despite the structural similarity shared between bacterial and fungal aromatic polyketides, fundamental differences in cyclization strategies exist. It was reported that a PT domain controls the cyclization steps of the nascent non-reducing (NR) fungal polyketides and frequently a thioesterase/Claisen-like cyclase (TE/CLC) is found at the C-terminus of the NR-PKSs to catalyze additional cyclization steps and product release (Zhou et al., 2010). There is limited data regarding cyclization and release processes in the biosynthesis of heterocyclic polyketides produced by fungal highly reducing PKSs (HR-PKSs), like OTA PKS. They are a subgroup of iterative type I fungal PKSs that produce linear, reduced polyketides in an assembly line process. The PKS intermediates remain bound to the megaenzyme via a thioester linkage during the whole process. Many HR-PKSs lack a C-terminal release domain, even though some HR-PKSs occur as fused bimodules with a C-terminal NRPS module, such as the well-studied lovastatin HR-PKS LovB which contains a C-terminal NRPS condensing (C) domain (Herbst et al., 2018). More often, cyclization is thought to happen spontaneously via intramolecular interactions within the growing polyketide. The alternative hypothesis could be the involvement of a cyclase gene separate from the PKS gene. In this regard, the biosynthetic pathway for aurovertin by the ascomycete *Calcarisporium arbuscula* is thought to involve a cyclase protein (aurE), with sequence homology to bacterial aromatic polyketide cyclase SnoaL, that may enhance (but is not essential for) pyrone formation and product release from the aurA PKS (Mao et al., 2015; Hang et al., 2016).

Concerning OTA biosynthetic pathway, the cyclization process that leads to the formation of the heterocyclic structure of OTβ, in the first stages, has remained unclear. The identification of a gene encoding a SnoaL like protein (*otaY*) in the OTA cluster suggests its putative role in the cyclization of OTA polyketide backbone.

Our comparative genomic, transcriptomic and phylogenomic analyses support inclusion of the *otaY* gene in the OTA biosynthetic gene cluster. Moreover, our results pointed out the need for future research to confirm the role of *otaY* gene in OTA biosynthesis by gene knock-out linked to the analysis of metabolic profile to track the intermediate metabolites accumulated in *otaY* defective strains compared to wild type strains.

## Data Availability Statement

The datasets presented in this study can be found in online repositories. The names of the repository/repositories and accession number(s) can be found below: https://www.ncbi.nlm.nih.gov/genbank/, MT706047; MT706048; and MT706049.

## Author Contributions

GP, MF, AG, and SB contributed to conception and design of the study. MF and DM performed the experiments and the analysis. AG and MF wrote the first draft of the manuscript and performed the genomic annotation. GP and DM performed the phylogenetic analysis. SB performed the comparative analysis. All authors contributed to the manuscript revision, read and approved the submitted version.

## Conflict of Interest

The authors declare that the research was conducted in the absence of any commercial or financial relationships that could be construed as a potential conflict of interest.

## Footnotes

1 http://jgi.doe.gov/fungi

2 http://1000.fungalgenomes.org

3 https://genome.jgi.doe.gov/portal

4 www.ispa.cnr.it/Collection

5 https://www.ebi.ac.uk/Tools/st/emboss_sixpack/

6 http://smart.embl-heidelberg.de/

7 http://tcoffee.crg.cat/apps/tcoffee/index.html

## Supplementary Material

Supplementary Figure 1 The highest log likelihood (−118408.77) tree obtained by RaxML analysis of 863 SnoAL domain is shown with bootstrap value. The yellow highlighted cluster represent the 21 SnoaL domains (red cluster) identified in the OTA biosynthetic clusters, plus the sister cluster of two no producing OTA species *A. cervinus* and *A. parvulus.*

## References

[B1] Baker S. E. Perrone G. Gallo A. Mulè G. Susca A. Logrieco A. F. (2009). “The Aspergillus carbonarius genome: Analysis of potential secondary metabolite biosynthetic gene clusters,” in*Proceedngs of the 25th Fungal Genetics Conference*, Asilomar, CA, 106.

[B2] Brakhage A. A. (2013). Regulation of fungal secondary metabolism. *Nat. Rev. Microbiol.* 11 21–32. 10.1038/nrmicro2916 23178386

[B3] Brakhage A. A. Schroeckh V. (2011). Fungal secondary metabolites - Strategies to activate silent gene clusters. *Fungal Genet. Biol.* 48 15–22. 10.1016/j.fgb.2010.04.004 20433937

[B4] Cacho R. A. Tang Y. Chooi Y. H. (2014). Next-generation sequencing approach for connecting secondary metabolites to biosynthetic gene clusters in fungi. *Front. Microbiol.* 5:774. 10.3389/fmicb.2014.00774 25642215 PMC4294208

[B5] Castegnaro M. Canadas D. Vrabcheva T. Petkova-Bocharova T. Chernozemsky I. N. Pfohl-Leszkowicz A. (2006). Balkan endemic nephropathy: role of ochratoxins A through biomarkers. *Mol. Nutr. Food Res.* 50 519–529. 10.1002/mnfr.200500182 16715544

[B6] Culebras M. P. V. Crespo-Sempere A. Gil J. V. Ramón D. (2009). Acyl transferase domains of putative polyketide synthase (PKS) genes in Aspergillus and Penicillium producers of ochratoxin A and the evaluation of PCR primers to amplify PKS sequences in black Aspergillus species. *Food Sci. Technol. Int.* 15 97–105. 10.1177/1082013208102743

[B7] de Oliveira D. Ocaña K. (2015). Parallel computing in genomic research: advances and applications. *Adv. Appl. Bioinforma. Chem*. 8 23–25. 10.2147/AABC.S64482 26604801 PMC4655901

[B8] el Khoury A. E. Atoui A. (2010). Ochratoxin a: general overview and actual molecular status. *Toxins* 2 461–493. 10.3390/toxins2040461 22069596 PMC3153212

[B9] Ferrara M. Perrone G. Gambacorta L. Epifani F. Solfrizzo M. Gallo A. (2016). Identification of a halogenase involved in the biosynthesis of ochratoxin A in Aspergillus carbonarius. *Appl. Environ. Microbiol.* 82 5631–5641. 10.1128/AEM.01209-16 27422838 PMC5007760

[B10] Frisvad J. C. Hubka V. Ezekiel C. N. Hong S. B. Nováková A. Chen A. J. (2019). Taxonomy of Aspergillus section Flavi and their production of aflatoxins, ochratoxins and other mycotoxins. *Stud. Mycol*. 93 1–63. 10.1016/j.simyco.2018.06.001 30108412 PMC6080641

[B11] Frisvad J. C. Larsen T. O. (2015). Chemodiversity in the genus Aspergillus. *Appl. Microbiol. Biotechnol.* 99 7859–7877. 10.1007/s00253-015-6839-z 26243055

[B12] Gallo A. Bruno K. Solfrizzo M. Perrone G. Mulè G. Visconti A. (2012). New insight in the ochratoxin A biosynthetic pathway by deletion of an nrps gene in Aspergillus carbonarius. *Appl. Environ. Microbiol*. 78 8208–8218. 10.1128/AEM.02508-2 22983973 PMC3497364

[B13] Gallo A. Ferrara M. Perrone G. (2017). Recent advances on the molecular aspects of ochratoxin A biosynthesis. *Curr. Opin. Food Sci.* 17 49–56. 10.1016/j.cofs.2017.09.011

[B14] Gallo A. Knox B. P. Bruno K. S. Solfrizzo M. Baker S. E. Perrone G. (2014). Identification and characterization of the polyketide synthase involved in ochratoxinA biosynthesis in Aspergillus carbonarius. *Int. J. Food Microbiol.* 179 10–17. 10.1016/j.ijfoodmicro.2014.03.013 24699234

[B15] Gil-Serna J. García-Díaz M. González-Jaén M. T. Vázquez C. Patiño B. (2018). Description of an orthologous cluster of ochratoxin A biosynthetic genes in Aspergillus and Penicillium species. A comparative analysis. *Int. J. Food Microbiol.* 268 35–43. 10.1016/j.ijfoodmicro.2017.12.028 29324288

[B16] Grigoriev I. V. Cullen D. Goodwin S. B. Hibbett D. Jeffries T. W. Kubicek C. P. (2011). Fueling the future with fungal genomics. *Mycology* 2, 192–209

[B17] Grigoriev I. V. Nikitin R. Haridas S. Kuo A. Ohm R. Otillar R. (2014). MycoCosm portal: gearing up for 1000 fungal genomes. *Nucl. Acids Res.* 42 D699–D704. 24297253 10.1093/nar/gkt1183 PMC3965089

[B18] Hang L. Liu N. Tang Y. (2016). Coordinated and Iterative Enzyme Catalysis in Fungal Polyketide Biosynthesis. *ACS Catal.* 6 5935–5945. 10.1021/acscatal.6b01559 28529817 PMC5436725

[B19] Herbst D. A. Townsend C. A. Maier T. (2018). The architectures of iterative type I PKS and FAS. *Nat. Prod. Rep.* 35 1046–1069. 10.1039/c8np00039e 30137093 PMC6192843

[B20] Houbraken J. Kocsubé S. Visagie C. M. Yilmaz N. Wang X-C. Meijer M. (2020). Classification of Aspergillus, Penicillium, Talaromyces and related genera (Eurotiales): an overview of families, genera, subgenera, sections, series and species. *Stud. Mycol.* 95 5–169. 10.1016/j.simyco.2020.05.002 32855739 PMC7426331

[B21] IARC (1993). *Monographs on the Evaluation of Carcinogenic Risks to Humans: Some Naturally Occurring Substances: Food Items and Constituents, Heterocyclic Aromatic Amines and Mycotoxins, International Agency for Research on Cancer.* France: IARC.

[B22] Jones D. T. Taylor W. R. Thornton J. M. (1992). The rapid generation of mutation data matrices from protein sequences. *Comp. Appl. Biosci.* 8 275–282. 10.1093/bioinformatics/8.3.275 1633570

[B23] Jørgensen K. (2005). Occurrence of ochratoxin A in commodities and processed food - A review of EU occurrence data. *Food Addit. Contam.* 22 26–30. 10.1080/02652030500344811 16332618

[B24] Katoh K. Standley D. M. (2013). MAFFT multiple sequence alignment software version 7: improvements in performance and usability. *Mol. Biol. Evol.* 30:772-780. 10.1093/molbev/mst010 23329690 PMC3603318

[B25] Khaldi N. Seifuddin F. T. Turner G. Haft D. Nierman W. C. Wolfe K. H. (2010). SMURF: genomic mapping of fungal secondary metabolite clusters. *Fungal Genet. Biol.* 47 736–741. 10.1016/j.fgb.2010.06.003 20554054 PMC2916752

[B26] Kjærbølling I. Vesth T. Frisvad J. C. Nybo J. L. Theobald S. Kildgaard S. (2020). A comparative genomics study of 23 Aspergillus species from section Flavi. *Nat. Commun.* 11:106. 10.1038/s41467-019-14051-y 32107379 PMC7046712

[B27] Kjærbølling I. Vesth T. C. Frisvad J. C. Nybo J. L. Theobald S. Kuo A. (2018). Linking secondary metabolites to gene clusters through genome sequencing of six diverse Aspergillus species. *Proc. Natl. Acad. Sci. U.S.A.* 115 E753–E761. 10.1073/pnas.1715954115 29317534 PMC5789934

[B28] Kumar S. Stecher G. Li M. Knyaz C. Tamura K. (2018). MEGA X: molecular evolutionary genetics analysis across computing platforms. *Mol. Biol. Med.* 35 1547–1549. 10.1093/molbev/msy096 29722887 PMC5967553

[B29] Malir F. Ostry V. Pfohl-Leszkowicz A. Malir J. Toman J. (2016). Ochratoxin A: 50 years of research. *Toxins* 8 12–15. 10.3390/toxins8070191 27384585 PMC4963825

[B30] Mao X. M. Zhan Z. J. Grayson M. N. Tang M. C. Xu W. Li Y. Q. (2015). Efficient biosynthesis of fungal polyketides containing the dioxabicyclo-octane ring system. *J. Am. Chem. Soc.* 137 11904–11907. 10.1021/jacs.5b07816 26340065 PMC4903023

[B31] Metzker M. L. (2010). Sequencing technologies - the next generation. *Nat. Rev. Genet.* 11 31–46. 10.1038/nrg2626 19997069

[B32] Miller M. A. Pfeiffer W. Schwartz T. (2010). Creating the CIPRES Science Gateway for inference of large phylogenetic trees. *Proceedings of the Gateway Computing Environments Workshop (GCE)*, New Orleans, LA, 1–8

[B33] Pel H. J. de Winde J. H. Archer D. B. Dyer P. S. Hofmann G. Schaap P. J. (2007). Genome sequencing and analysis of the versatile cell factory Aspergillus niger CBS 513.*88*. *Nat. Biotechnol.* 25 221–231. 10.1038/nbt1282 17259976

[B34] Ronquist F. Teslenko M. Van Der Mark P. Ayres D. L. Darling A. Höhna S. (2012). MrBayes 3.2: efficient Bayesian phylogenetic inference and model choice across a large model space. *Syst. Biol*. 61 539–542. 10.1093/sysbio/sys029 22357727 PMC3329765

[B35] Samson R. A. Visagie C. M. Houbraken J. Hong S. B. Hubka V. Klaassen C. H. W. (2014). Phylogeny, identification and nomenclature of the genus Aspergillus. *Stud. Mycol*. 78 141–173. 10.1016/j.simyco.2014.07.004 25492982 PMC4260807

[B36] Sidhu G. S. (2002). Mycotoxin genetics and gene clusters. *Eur. J. Plant Pathol.* 108 705–711. 10.1023/A:1020613413483

[B37] Stamatakis A. (2014). RAxML Version 8: a tool for Phylogenetic Analysis and Post-Analysis of Large Phylogenies. *Bioinformatics.* 30 1312–1313 10.1093/bioinformatics/btu033 24451623 PMC3998144

[B38] Sultana A. Kallio P. Jansson A. Wang J. Niemi J. Mäntsälä P. (2004). Structure of the polyketide cyclase SnoaL reveals a novel mechanism for enzymatic aldol condensation. *EMBO J.* 23 1911–1921. 10.1038/sj.emboj.7600201 15071504 PMC404321

[B39] Vesth T. C. Nybo J. L. Theobald S. Frisvad J. C. Larsen T. O. Nielsen K. F. (2018). Investigation of inter- and intraspecies variation through genome sequencing of Aspergillus section Nigri. *Nat. Genet*. 50 1688–1695. 10.1038/s41588-018-0246-1 30349117

[B40] Wang Y. Wang L. Wu F. Liu F. Wang Q. Zhang X. (2018). A Consensus Ochratoxin A Biosynthetic Pathway: Insights from the Genome Sequence of Aspergillus ochraceus and a Comparative Genomic Analysis. *Appl. Environ. Microbiol.* 84 e1009–e1018. 10.1128/AEM.01009-18 30054361 PMC6146979

[B41] Zhou H. Li Y. Tang Y. (2010). Cyclization of aromatic polyketides from bacteria and fungi. *Nat. Prod. Rep.* 27 839–868. 10.1039/b911518h 20358042 PMC2884966

