# Supplementary figures and images for: Comparative Genomic Analysis of Ochratoxin A Biosynthetic Cluster in Producing Fungi: New Evidence of a Cyclase Gene Involvement
Source: Front Microbiol. 2020 Dec 18;11:581309. doi: 10.3389/fmicb.2020.581309 (PMC7775548; doi:10.3389/fmicb.2020.581309)

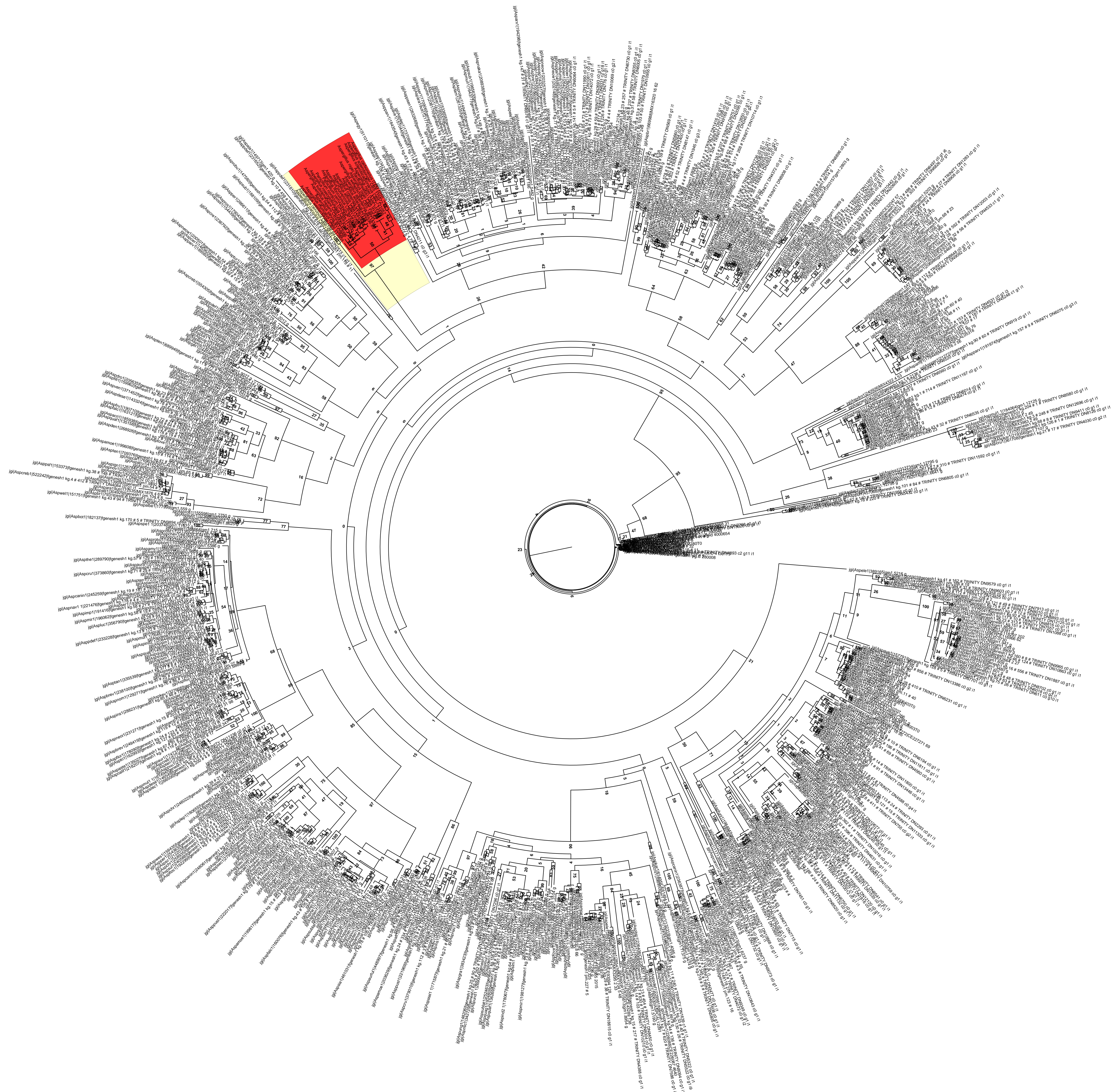

Supplement: Supplementary Figure 1 — The highest log likelihood (−118408.77) tree obtained by RaxML analysis of 863 SnoAL domain is shown with bootstrap value. The yellow highlighted cluster represent the 21 SnoaL domains (red cluster) identified in the OTA biosynthetic clusters, plus the sister cluster of two no producing OTA species A. cervinus and A. parvulus. [file Data_Sheet_1.PDF]
